# Diversity of Rare and Abundant Prokaryotic Phylotypes in the Prony Hydrothermal Field and Comparison with Other Serpentinite-Hosted Ecosystems

**DOI:** 10.3389/fmicb.2018.00102

**Published:** 2018-02-06

**Authors:** Eléonore Frouin, Méline Bes, Bernard Ollivier, Marianne Quéméneur, Anne Postec, Didier Debroas, Fabrice Armougom, Gaël Erauso

**Affiliations:** ^1^Aix-Marseille Univ, Université de Toulon, CNRS, IRD, MIO UM 110, Marseille, France; ^2^CNRS UMR 6023, Laboratoire “Microorganismes – Génome et Environnement”, Université Clermont Auvergne, Clermont-Ferrand, France

**Keywords:** microbial communities, prony, shallow hydrothermal field, alkaliphiles, methanosarcinales, serpentinization

## Abstract

The Bay of Prony, South of New Caledonia, represents a unique serpentinite-hosted hydrothermal field due to its coastal situation. It harbors both submarine and intertidal active sites, discharging hydrogen- and methane-rich alkaline fluids of low salinity and mild temperature through porous carbonate edifices. In this study, we have extensively investigated the bacterial and archaeal communities inhabiting the hydrothermal chimneys from one intertidal and three submarine sites by 16S rRNA gene amplicon sequencing. We show that the bacterial community of the intertidal site is clearly distinct from that of the submarine sites with species distribution patterns driven by only a few abundant populations, affiliated to the *Chloroflexi* and *Proteobacteria* phyla. In contrast, the distribution of archaeal taxa seems less site-dependent, as exemplified by the co-occurrence, in both submarine and intertidal sites, of two dominant phylotypes of *Methanosarcinales* previously thought to be restricted to serpentinizing systems, either marine (Lost City Hydrothermal Field) or terrestrial (The Cedars ultrabasic springs). Over 70% of the phylotypes were rare and included, among others, all those affiliated to candidate divisions. We finally compared the distribution of bacterial and archaeal phylotypes of Prony Hydrothermal Field with those of five previously studied serpentinizing systems of geographically distant sites. Although sensu stricto no core microbial community was identified, a few uncultivated lineages, notably within the archaeal order *Methanosarcinales* and the bacterial class *Dehalococcoidia* (the candidate division MSBL5) were exclusively found in a few serpentinizing systems while other operational taxonomic units belonging to the orders *Clostridiales, Thermoanaerobacterales*, or the genus *Hydrogenophaga*, were abundantly distributed in several sites. These lineages may represent taxonomic signatures of serpentinizing ecosystems. These findings extend our current knowledge of the microbial diversity inhabiting serpentinizing systems and their biogeography.

## Introduction

Serpentinization is the alteration process that abiotically transforms olivine and pyroxene-rich rocks into serpentinites and yields alkaline hot fluids enriched in H_2_ and CH_4_ (Chavagnac et al., 2013). Active serpentinization generates physico-chemical conditions that might have prevailed in early Earth and could have been fundamental with regard to the origin of life (Russell et al., 2010; Schrenk et al., 2013). Several serpentinizing systems, particularly terrestrial ones, have recently been studied by molecular approaches using next generation sequencing, to reveal the indigenous microbial community. They included the Cabeço de Vide Aquifer (CVA, Portugal) (Tiago and Veríssimo, 2013) and the ophiolites of Tablelands (Canada) (Brazelton et al., 2013), Santa Elena (Costa Rica) (Sánchez-Murillo et al., 2014; Crespo-Medina et al., 2017), Zambales (Philippines) (Woycheese et al., 2015), Samail (Oman) (Rempfert et al., 2017), Chimaera (Turkey) (Neubeck et al., 2017), Voltri (Italy) (Quéméneur et al., 2015; Brazelton et al., 2016), The Cedars (Placer, CA, United States) (Suzuki et al., 2017) and the nearby site of Coast Range Ophiolite Microbial Observatory (CROMO, Burbank, CA, United States) (Twing et al., 2017). In contrast, only a few submarine serpentinizing fields have been investigated using high-throughput sequencing. Their sampling is indeed complicated due to their remote geographic location and great depths. Among them, the emblematic Lost-City hydrothermal field (LCHF) was the only serpentinizing ecosystem to be subjected to microbiological study targeting the rare biosphere (Brazelton et al., 2010).

The recently described Prony Hydrothermal Field (PHF) (Pelletier et al., 2006; Monnin et al., 2014; Quéméneur et al., 2014; Postec et al., 2015) is a shallow (50 m maximum depth) marine alkaline hydrothermal system, located in the Bay of Prony, South of New Caledonia. The run-off waters from the rocky hills surrounding the Bay percolate through the densely fractured peridotite basement. The resulting end-member fluids discharge into the lagoon and, upon mixing with ambient seawater, precipitate, forming large carbonate edifices resembling Lost-City chimneys (Monnin et al., 2014). Similarly, to those of Lost-City, PHF fluids are characterized by high pH values (between 9.0 and 11.5) and elevated N_2_, H_2_, and CH_4_ concentrations (Monnin et al., 2014). However, the PHF fluids are different in terms of temperatures (maximum 40°C) and salinity (less than 0.5 g/L). The microbial community composition of the inner part of PHF chimneys has been previously characterized by culture-dependent (Ben Aissa et al., 2014, 2015; Mei et al., 2014; Bes et al., 2015), molecular-based techniques such as fingerprinting methods (SSCP and DGGE), clonal Sanger sequencing (Quéméneur et al., 2014; Postec et al., 2015), and Fluorescent *In Situ* Hybridization (FISH) and micro-imaging (Pisapia et al., 2017). Molecular-based studies revealed that bacteria largely outnumbered the archaea communities. The bacterial communities were composed of diverse populations mostly affiliated to the *Chloroflexi* and *Firmicutes* in the submarine sites and to the *Proteobacteria* in the intertidal sites (Quéméneur et al., 2014). The low-diversity archaeal communities were dominated by *Methanosarcinales* with phylotypes similar to those of the Lost-City *Methanosarcinales* (LCMS) and The Cedars *Methanosarcinales* (TCMS). While previous studies in PHF have identified the most abundant phylotypes, a substantial part of the indigenous microbial diversity was assumed to remain missing from the 16S rRNA genes inventories, due to the low-coverage sequencing (Quéméneur et al., 2014; Postec et al., 2015). Indeed, the coverage of the clone libraries was low, especially for the bacteria, with values ranging from 54 to 79%. Since such approaches underestimate the true biodiversity of environments (Curtis and Sloan, 2005), higher resolution techniques such as deep sequencing are therefore required in order to recover the overall microbial diversity of PHF. A preliminary study was performed with next-generation sequencing in PHF sites, but was only focused on the diversity of potentially hydrogen-producing bacteria and their hydrogenases (Mei et al., 2016).

In this study, we performed 454 pyrosequencing of the V1–V3 regions of the prokaryotic 16S rRNA gene in order to investigate the composition and the diversity of the microbial biosphere inhabiting carbonate chimneys of four sampling sites of the PHF. We showed that the diversity of bacteria (but not archaea) was site-specific and that the overall PHF biosphere was dominated by rare prokaryotic phylotypes. Finally, using publicly available sequences of 16S rRNA genes, we reported the co-occurrence of bacterial and archaeal operational taxonomic units (OTUs) identified in PHF and in other serpentinizing systems.

## Materials and Methods

### Sample Collection

Description of the sampling sites, sample processing and storage, and physico-chemical characteristics of PHF have been reported previously (Monnin et al., 2014; Quéméneur et al., 2014; Postec et al., 2015). Briefly, four active carbonate chimneys were sampled at four distinct sites of the Bay of Prony, including the “Bain des Japonais” (BdJ), the sites (ST) ST07 (also called “Aiguille de Prony”), ST09 and ST12. The BdJ site is located on the foreshore of the Carenage Bay and is uncovered at low tide. It consists of many fluid outlets building a carbonate plateau. There, small needle-like brittle structures of 10–20 cm high and 3–6 cm in diameter were collected. The submarine sites ST07, ST12, and ST09, located in the Prony Bay, were sampled at 16 meters below sea level (mbsl), 38 mbsl, and 48 mbsl, respectively. They form large carbonate edifices, carrying several chimneys of up to several meters high. The chimneys are porous structures, mainly composed of brucite [Mg(OH)_2_], aragonite and calcite (CaCO_3_) and magnesium carbonates (MgCO_3_) (Pisapia et al., 2017). The external walls of the mature submarine chimneys are colonized by various invertebrates (e.g., ascidia, sponges, corals). The top of such chimneys was collected by scuba-diving in each submarine site. They were kept at 4–8°C during their transportation (∼3 h) to the laboratory, where they were immediately processed. Cross sections (4 cm thick) of these chimneys top were realized aseptically; peripheral parts of the sections (2 cm thick) were cautiously removed using a chisel and sub-samples of the inside parts of the chimneys, bathed with the alkaline fluid, were collected and stored at –80°C until DNA extraction, as detailed previously (Quéméneur et al., 2014; Postec et al., 2015).

### Ribosomal RNA Gene Amplification and 454 Pyrosequencing

DNA extraction and quantification procedures have been described previously (Postec et al., 2015). Briefly, about 0.4 g of chimney slurry was transferred into a sterile 2 mL tube containing glass beads (lysing matrix E from MP BioMedicals) and 0.8 mL of lysis buffer (Tris.HCl 100 mM, NaCl 100 mM, EDTA 50 mM, pH 8). Cell lysis was achieved with chemical reactions (adding Sodium Dodecyl Sulfate and Lauryl Sarcosine) and mechanic disruption using a Fast Prep homogenizer. DNA was extracted using phenol:chloroform:isoamyl alcohol and chloroform:isoamyl alcohol. DNA from each sample or dilution was used as template for 16S rRNA amplification. The hypervariable region V1–V3 of the 16S rRNA gene was amplified using the pair of primers 27F (5′-AGAGTTTGATCCTGGCTCAG-3′)/533R (5′-TTACCGCGGCTGCTGGCAC-3′) and 21F (5′-TCYGKTTGATCCYGSCRGA-3′)/529R (5′-TCGCGCCTGCTGCRCCCCGT-3′) for bacteria and archaea, respectively. The forward and reverse primers were barcoded (Supplementary Table S1). PCR reactions were performed in a 50 μl mixture containing Platinium^TM^ *Taq* High Fidelity (Life Technologies) 1 unit, High Fidelity PCR Buffer 1X, dNTP mixture 0.2 mM each, MgSO4 2 mM, primer mix 0.3 μM each, and 2 μl template DNA. The protocol mentioned below was used for amplification: initial denaturation at 95°C for 2 min, followed by 25 cycles of denaturation 95°C for 45 s, primers annealing at 54°C for 45 s, elongation at 72°C for 1 min 30 s, and a final elongation step at 72°C for 7 min. At least three PCR products from each combination of sample and primer pairs were pooled and then purified with QIAquick Gel Extraction Kit (Qiagen). An equimolar amount of each PCR pool was finally used for library construction (addition of the adaptors by ligation) and pyrosequencing using 454 GS FLX Titanium technology (Beckman Coulter Genomics).

### 16S rRNA Gene Analysis

Pyrosequencing of the V1–V3 region of the microbial 16S rRNA generated 188,058 sequences. Raw nucleotide sequence data were submitted to the European Nucleotide Archive under project accession number PRJEB21795. These sequences were filtered according the following criteria: read length between 150 and 500 bp, no ambiguous bases, average quality score ≥25, and no error allowed in primer and barcode sequences. A total of 74,457 and 95,916 high quality sequences were obtained for the bacteria and archaea, respectively. Alignments and clustering to Operational Taxonomic Units (OTUs) were carried out using the QIIME 1.9.1 workflow (http://www.qiime.org/) (Caporaso et al., 2010b). Chimera sequences were identified and removed using UCHIME (Edgar et al., 2011). Multiple sequence alignments were performed using PyNAST (Caporaso et al., 2010a). OTUs were defined by clustering sequences using UCLUST (Edgar, 2010) with a pairwise distance threshold value of 3%, a cutoff currently used for species demarcation while reducing the potential inflation of the number of OTUs due to pyrosequencing errors. To limit the impact of spurious OTUs, low abundance OTUs accounting for less than 0.005% of total sequences were removed as recommended by Bokulich et al. (2013). Taxonomic assignment of the filtered sequences was performed using the RDP classifier algorithm (Wang et al., 2007) with a minimum bootstrap confidence of 80% against the SILVA database (Quast et al., 2013) release for QIIME (v.123). The OTU table was rarefied (i.e., downsampled to the sample with the smallest set of sequences) to reduce sequencing depth heterogeneity between samples. There is currently no standardized threshold for the rare biosphere. Here, rare OTUs were defined as OTUs comprising 0.005 to 0.2% of sequences per sample, an empirical threshold previously adopted by Hugoni et al. (2013). OTUs comprising 0.2 to 1% of sequences per sample were considered as intermediate and OTUs representing more than 1% of sequences per sample were considered as abundant. The alpha diversity within the four samples was estimated using the Shannon (Shannon and Weaver, 1949) and Simpson (Simpson, 1949) indices, and the beta diversity among the samples was measured using weighted UniFrac (Lozupone and Knight, 2005) metric. Taxonomic composition, and diversity results were visualized using the R package phyloseq v.1.14.0 (McMurdie and Holmes, 2013). BLASTn (Basic Local Alignment Search Tool) searches against the NCBI (National Center for Biotechnology Information) database of nucleotides (nt) and against the RefSeq database were performed for some OTU sequences. To infer the phylogeny of *Methanosarcinales*, cultivated members of this order and environmental clones were added to the sequences of abundant or ubiquitous PHF OTUs. The V1–V3 regions of these sequences were aligned with MAFFT v7.123b (Katoh et al., 2002) and a maximum likelihood tree was inferred using the software SeaView (Gouy et al., 2010) with 1000 bootstrapped trials.

### Taxonomic Comparison of Phylotypes in Serpentinizing Environments

The correctly formatted amplicons and clone libraries of five serpentinizing ecosystems were downloaded from public databases (SRA and Genbank). These data constituted a subset of known serpentinizing systems, including four continental environments: Cabeço de Vide Aquifer (Tiago and Veríssimo, 2013), The Cedars (Suzuki et al., 2013), Voltri ophiolite (Quéméneur et al., 2015), Leka ophiolite (Daae et al., 2013), and one submarine system: the famous Lost-City Hydrothermal Field (Schrenk et al., 2004; Brazelton et al., 2006). A closed-reference OTU picking process was carried out to compare the sequences of amplicons and clone libraries covering different regions of the 16S rRNA gene. The sequences were clustered against a reference sequence database (SILVA database, v123). The OTUs shared among samples were visualized with Anvi’o v2.3.2 (Eren et al., 2015).

## Results and Discussion

### Microbial Community Diversity

The bacterial species richness of the sampling sites comprised 1,979 distinct OTUs (Supplementary Figure S1A). However, the species richness distribution varied significantly from one site to another, with at least a twofold decrease in OTU abundance for BdJ, ST07, and ST09 compared to the ST12 site (1203 OTUs, Supplementary Table S2). The presence of more than 780 OTUs, specific to the ST12 site (Supplementary Figure S1A) and comprising mainly of rare taxa, might result from a lower in situ pH condition (around 9.2), in comparison with the other studied PHF sites (pH > 9.8) (Monnin et al., 2014). This trend has previously been observed in the Coast Range Ophiolite serpentinizing site, where circumneutral pH wells contained many rare taxa that were absent in the high pH ones (Twing et al., 2017), most likely because these latter drastic conditions selected the few most alkaliphilic members within the prokaryotic community. The overall species richness was considerably lower in the archaea (310 OTUs) than in the bacteria, and also showed an imbalance in OTU distribution across the four sites (Supplementary Figure S1B and Supplementary Table S2). The Shannon and Simpson indexes indicated lower archaeal diversity, as compared to the bacterial one (Supplementary Figure S2 and Supplementary Table S2). This lower richness and diversity of archaea is not specific to PHF as it has been generally observed in various environments globally (Aller and Kemp, 2008), including deep-sea hydrothermal vents (Anderson et al., 2015) and Lost-City (Brazelton et al., 2010). The rarefaction curves had a near-asymptotic shape (Supplementary Figure S3) suggesting that the sequencing effort was suitable to capture the near-full diversity of this serpentinizing environment.

### Bacterial Phylogenetic Composition in PHF

**FIGURE 1:**
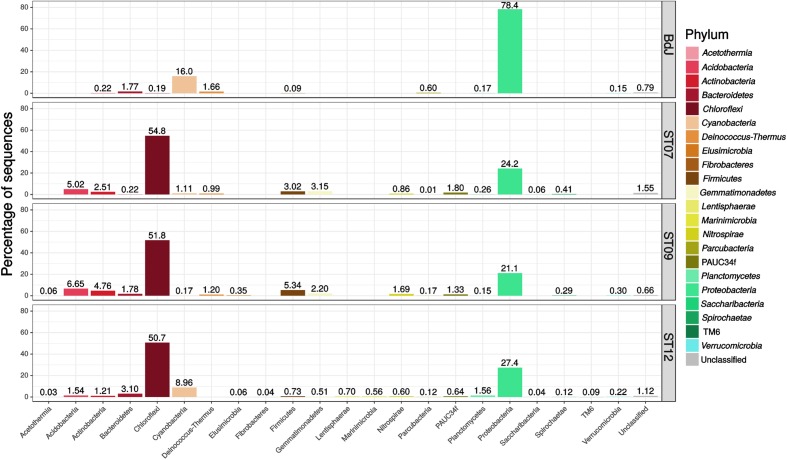
Phylum-level bacterial diversity in Prony Hydrothermal Field (PHF) sites based on 16S rRNA gene sequences (V1–V3 region). The relative abundances were estimated for the four chimneys collected in BdJ, ST07, ST09, and ST12 sites.

The bacterial community composition of the PHF sites encompassed 22 different phyla (including six candidate phyla) reflecting a high biodiversity. The phylum *Proteobacteria* was preponderant in the intertidal BdJ (78.4% of sequences, 244 OTUs), while the three submarine sites were mainly composed of *Chloroflexi* (50.7–54.8%, 150–161 OTUs) and, to a lesser extent, of *Proteobacteria* members (21.1–27.4%, 250–603 OTUs) (**Figure 1**). Remarkably, all other bacterial phyla shared by all the PHF sites were essentially recovered at much lower abundance. These bacterial phyla comprised *Cyanobacteria* (0.2–16.0%), *Actinobacteria* (0.2–4.8%), *Bacteroidetes* (0.2–3.1%), *Firmicutes* (0.1–5.3%), *Planctomycetes* (0.2–1.6%), and the candidate phylum *Parcubacteria* (formerly candidate division OD1) (0.01–0.6%). By contrast, the *Acetothermia*, *Acidobacteria, Gemmatimonadetes*, and *Nitrospirae* phyla were only retrieved from submarine sites, representing 7.4% of the total sequences. Relative abundances of some bacterial phyla detected in our study were not in agreement with those reported by sequencing clone libraries (Quéméneur et al., 2014; Postec et al., 2015). This was the case for the *Firmicutes* and the *Parcubacteria*, which were found in much lower proportion in this study. Nevertheless, significant shifts in phyla proportions have already been observed in the same chimney edifice over a 6 year survey (Postec et al., 2015). Our pyrosequencing approach also allowed the identification of 6 novel phyla that had not been previously detected. Members of all these phyla, i.e., *Fibrobacteres*, *Lentisphaerae*, *Marinimicrobia*, PAUC34f, *Saccharibacteria*, and *Verrucomicrobia*, were found among the large diversity of the ST12 site. On the contrary, several phyla previously found in PHF: *Atribacteria*, *Microgeomates, Omnitrophica*, and candidate division NPL-UPA2 (Quéméneur et al., 2014; Postec et al., 2015; Mei et al., 2016; Pisapia et al., 2017) were not represented in this study. This discrepancy may be explained by the use of different PCR primers targeting different regions of the 16S rRNA gene of bacteria and archaea. It may also be the consequence of the ongoing evolution of the new generation sequencing technology (i.e., 454 Roche vs. Illumina). That raises a major issue regarding the lack of standards for 16S rRNA gene surveys, which reduces the reproducibility and complicates comparisons between studies (Escobar-Zepeda et al., 2015). Additionally, the sampling area of the chimneys (either central or more peripheral) could also explain these taxonomic discrepancies. Indeed, the highly porous structure of the chimney interiors is exposed to steep physicochemical gradients (in terms of salinity, pH, redox potential, nutrients) along their transverse axis, defining microhabitats colonized by contrasted microbial communities.

### Archaeal Phylogenetic Composition in PHF

**FIGURE 2:**
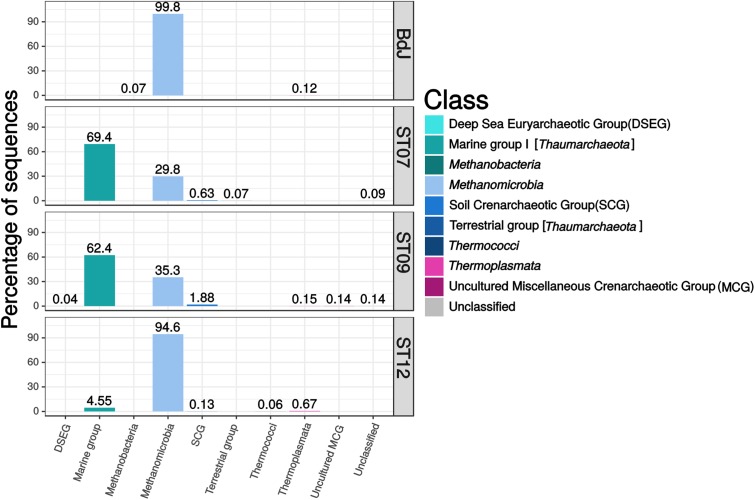
Class-level archaeal diversity in PHF sites based on 16S rRNA gene sequences (V1–V3 region). The relative abundances were estimated for the four chimneys collected in BdJ, ST07, ST09, and ST12 sites.

**FIGURE 3:**
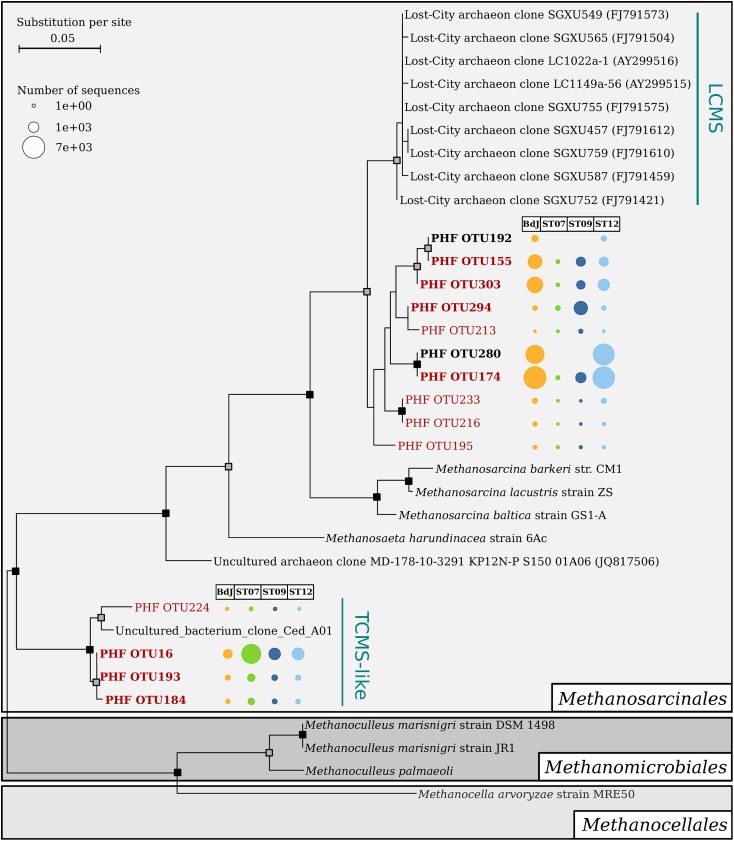
Maximum likelihood phylogenetic tree of V1–V3 archaeal 16S rRNA sequences affiliated to the order *Methanosarcinales*. Bootstrap support values are indicated on nodes with a gray or black square for support greater than 70% or 90%, respectively. Scale bar indicates substitutions per site. The *Methanosarcinales* sequences are marked in bold if they were abundant in at least one site of PHF, and red font color indicates ubiquitous operational taxonomic units (OTUs; found in the four chimneys of PHF). The bubble chart corresponds to the contingency table of OTUs. Uncultured *Methanosarcinales* clusters are abbreviated as follows: LCMS, Lost-City Methanosarcinales, and TCMS, The Cedars Methanosarcinales.

The archaeal community of the PHF sites mainly comprised potential methanogens and autotrophic ammonia-oxidizer representatives among *Euryarchaeota* and *Thaumarchaeota* phyla, respectively. The BdJ and ST12 sites were dominated by members of *Methanosarcinales* (99.8% of sequences, 38 OTUs and 94.6%, 44 OTUs, respectively) within the class *Methanomicrobia* (**Figure 2**). All abundant and ubiquitous OTUs of *Methanosarcinales* were similar to the sequences from other serpentinite-hosted ecosystems, as shown by OTU-based phylogeny (**Figure 3**). The first set of OTUs constituted a sister group of Lost-City *Methanosarcinales* (LCMS) (Schrenk et al., 2004) whereas the remaining OTUs were closely associated with the sequence of The Cedars *Methanosarcinales* (TCMS) (phylotype Ced_A01) (Suzuki et al., 2013). Although the present study reported a more accurate diversity than the previous studies based on clone libraries, no new phylotypes were identified within the *Methanosarcinales* group. Interestingly, although first detected in a terrestrial serpentinizing system (The Cedars), TCMS-like archaea were detected at lower abundance in the intertidal BdJ site than in the submarine sites of PHF. Moreover, here the overall abundance of the LCMS-like was two-fold higher than the abundance of the TCMS-like (**Figure 3**). This result is in opposition with the ratio previously observed in clone libraries and thus contradicts the hypothesis that the predominance of TCMS-like archaea in PHF was due to the low salinity of fluids (of meteoric origin such as in The Cedars) (Postec et al., 2015). Finally, these two specific phylotypes of *Methanosarcinales* were ubiquitous in all PHF sites. In contrast, the ST07 and ST09 sites were dominated by the *Thaumarchaeota* Marine Group I (69.4%, 179 OTUs and 62.4%, 194 OTUs, respectively), which was detected in all submarine sites despite its low abundance in the ST12 site (**Figure 2**). The Marine Group I is frequently observed in the global ocean (Auguet et al., 2010; Fernàndez-Guerra and Casamayor, 2012), which may explain why they are found only in submarine sites of PHF. In addition, the *Thaumarchaeota* Soil Crenarchaeotic Group was also detected in the submarine sites at very low-abundance level (0.1 to 1.9%, 3 to 6 OTUs) and may originate from the surrounding hills having been carried away by run-off waters. Other less represented archaeal classes belonged to the *Thermoplasmata* (mainly Marine Group II) (0.1–0.7%, 7–18 OTUs), *Methanobacteria* (0.1%, 2 OTUs at BdJ), Terrestrial Group of *Thaumarchaeota* (0.1%, 2 OTUs in ST07) and presumed thermophiles of the *Thermococci* class (0.1%, 1 OTU at ST12) (**Figure 2**). Finally, the remaining OTUs were affiliated to the Deep Sea Euryarchaeotic Group within the *Aenigmarchaeota* phylum and to the Miscellaneous Crenarchaeotic Group that were not previously detected in PHF. The archaea of the Miscellaneous Crenarchaeotic Group are widespread in marine sediment and are essentially retrieved from anoxic environments such as deep oceanic subsurface sediments or mud volcanoes (Kubo et al., 2012). Since the temperature of PHF fluids measured at the chimney outlets did not exceed 43°C, the presence of archaea closely related to hyperthermophilic species (e.g., *Thermococcus* spp.) originating from deep-sea environments was surprising. Despite their low abundance, such representatives of the *Thermococcales* were, however, previously detected in PHF, even in clone libraries, and the high GC content of their sequence strongly suggested they were bona fide hyperthermophiles (Postec et al., 2015). It is thus likely that they originated from subsurface hyperthermophilic microbial communities fed by serpentinization reactions and carried up to the surface by the fluid as previously suggested (Postec et al., 2015).

### Beta-Diversity and Abundant OTUs across the PHF Sites

**FIGURE 4:**
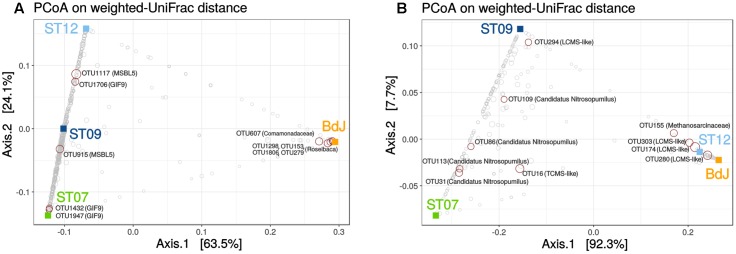
Beta diversity of bacterial **(A)** and archaeal **(B)** communities of PHF. PCoA analysis was carried out using the weighted UniFrac metric. Squares indicate PHF sites and circles correspond to OTUs. Taxonomic annotation was added to the top ten OTUs explaining this partition (red circles). Lost-City Methanosarcinales and The Cedars Methanosarcinales are abbreviated as LCMS and TCMS, respectively.

The bacteria taxonomic profiles observed for the three submarine sites were very similar, while the one observed for the intertidal site (BdJ) differed significantly (**Figure 4A**). This pattern of distribution, previously observed in a study on PHF (Quéméneur et al., 2014), may be explained by the influence of the marine environment regime (intertidal vs. submarine) variation in the composition of the end-member fluids (Monnin et al., 2014) or the resulting differences observed in the mineralogy of the carbonate chimneys (Pisapia et al., 2017). The relationship between species and sampling sites was mainly driven by a few dominant OTUs in the ordination plot (**Figure 4A**). Among them, five abundant OTUs in submarine sites were assigned to the class *Dehalococcoidia* (DEH, phylum *Chloroflexi*) and were related to clones retrieved from the serpentinizing springs of The Cedars (Suzuki et al., 2013). Two of these dominant OTUs (#915 and #1117) were assigned to the candidate order MSBL5 (Mediterranean Sea Brine Lake group 5) and three (#1432, #1706, and #1947) to the division GIF9 (Groundwater InFlow clone 9) (Supplementary Table S3). The candidate orders MSBL5 and GIF9 were originally detected in a deep-sea anoxic hypersaline lake (Daffonchio et al., 2006) and in a contaminated groundwater-fed bio reactor (Alfreider et al., 2002), respectively. These five dominant OTUs are quite divergent from any cultivated species (the closest similarity is found for OTU1947 with *Dehalogenimonas alkenigignens*, at 82% identity). The metabolic capabilities of these candidate groups are still largely unknown. Even for the entire class *Dehalococcoidia*, our knowledge of metabolism is essentially limited to that of the few cultivated representatives, which have the ability to oxidize hydrogen and reduce organohalogenic compounds (Kaster et al., 2014; Biderre-Petit et al., 2016). However, a single cell sequencing of a *Dehalococcoidia* phylotype, abundant in marine sediments, suggested that this bacterium may replace organohalides by dimethyl sulfoxide as a terminal electron acceptor (Wasmund et al., 2013). In the BdJ site, 4 OTUs (#1298, #153, #1806, #279) representing more than a third of all sequences (**Figure 4A**) were assigned to the haloalkaliphilic genus *Roseibaca* (*Alphaproteobacteria*), a sister genus of the phototrophic anaerobic sulfur-oxidizing *Rhodobaca*, previously detected as dominant in BdJ (Mei et al., 2016). Representatives of the *Roseibaca* genus are known to be rose-colored (Labrenz et al., 2009) and could in part explain the dark rose color of the biofilm covering the exteriors of BdJ chimneys. The BdJ site also contained two dominant OTUs (#607 and #763) affiliated to the *Comamonadaceae* family (*Betaproteobacteria*) showing 98% identity with *Hydrogenophaga defluvii* (Supplementary Table S3). Members of the genus *Hydrogenophaga* are chemoorganotrophic or chemolithoautotrophic, thriving by oxidation of hydrogen under microaerophilic conditions (Willems, 2014). Despite the potential involvement of *Firmicutes* in the organomineralization of carbonates chimneys in PHF (Pisapia et al., 2017), only one abundant OTU of this phylum was identified in submarine sites and assigned to uncultured anaerobic *Syntrophomonadaceae*. This OTU, however, differed from the one (KM207235 HPst091-1-1) reported to be dominant in juvenile chimneys of PHF (Pisapia et al., 2017), most likely because of the differences of age of the chimneys studied (i.e. mature vs. juvenile). Members of this family are anaerobes that usually grow in syntrophic association with hydrogenotrophic and formate-utilizing methanogens by thriving energy from oxidation of carboxylic acids (Sobieraj and Boone, 2006). These anaerobic oxidations are thermodynamically feasible only when the H_2_ or formate produced is concomitantly consumed by methanogens or other syntrophic partner. Recently, syntrophic associations were shown to permit the conversion of organic acids and alcohols to methane under extremely haloalkaline conditions (Sorokin et al., 2016). In PHF, such syntrophic associations between uncultivated members of the *Syntrophomonadaceae* and the *Methanosarcinales* could be a relevant strategy to overcome energetic limitations, due to the high pH conditions and the lack of electron acceptors.

For archaea, no differential segregation of OTUs between intertidal and submarine sites could be observed, in contrast to the trend previously reported (Quéméneur et al., 2014). Nevertheless, the beta-diversity analysis linked BdJ to ST12 (**Figure 4B**), with both sites harboring abundant OTUs related to the LCMS (their annotation was refined through phylogenetic analysis, **Figure 3**), whereas ST09 clustered with ST07 because they shared abundant OTUs related to Candidatus *Nitrosopumilus* (*Thaumarchaeota*) and numerous OTUs of Marine Group I (**Figure 4B** and Supplementary Table S4). In light of these beta-diversity results, the archaeal community seemed less site-dependent than the bacterial one, as exemplified by the two groups of *Methanosarcinales* OTUs (LCMS and TCMS) found in both intertidal and submarine serpentinizing systems of PHF sites.

### Comparison between Phylotypes of Serpentinizing Environments

A comparison of microbial communities across six geographically distant serpentinizing systems was carried out at the OTU level (at 97% identity threshold) used as a proxy of the prokaryotic species that is the most commonly found unit in microbial biogeographic studies. For practical reasons, this comparison was limited to the fraction of known microorganisms (i.e., those which could be assigned to a taxon in the Silva database), thus the results cannot be extrapolated to the whole prokaryotic community. Despite this limitation, some biogeographic patterns could be observed. The general OTU composition appeared to be quite different from one serpentinizing system to another and no OTU was common to all sites, but several related phylotypes were recovered from more than two systems; among them, a few were detected exclusively in serpentinizing systems. The distribution pattern of PHF phylotypes in other geographic sites and its implications is discussed below.

#### OTUs Belonging to Abundant Taxa Commonly Detected in Serpentinite-Hosted Ecosystems

**FIGURE 5:**
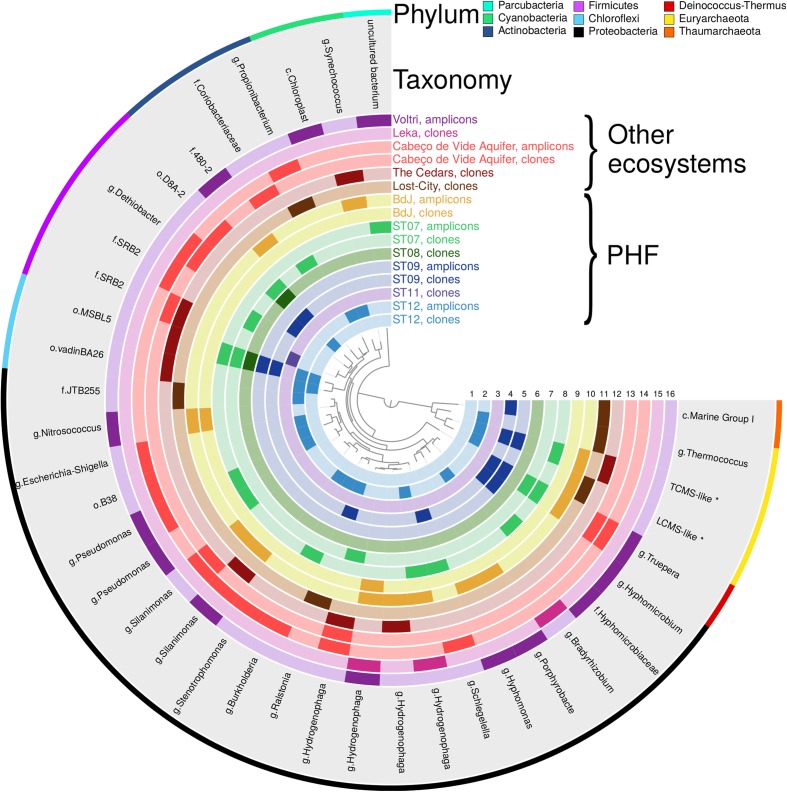
Presence-absence plot of OTUs shared at least by one sample of PHF sites and one sample from another serpentinizing ecosystem. The inner circles, numbered from 1 to 16, correspond to the different samples. The 13 samples of PHF include the circles 2, 5, 8, 10 (this study) and 1, 3, 4, 6, 7, 9 (Quéméneur et al., 2014; Postec et al., 2015; Pisapia et al., 2017). The seven other serpentinizing samples comprise the circles 11 from Lost-City Hydrothermal Field (Schrenk et al., 2004; Brazelton et al., 2006), 12 from The Cedars (Suzuki et al., 2013), 13, 14, from Cabeço de Vide (Tiago and Veríssimo, 2013), 15 from Leka ophiolite (Daae et al., 2013), and 16 from Voltri ophiolite (Quéméneur et al., 2015). Each circle color corresponds to a sampling system and the darker cells indicate the presence of OTUs. The taxonomic affiliation of OTUs is indicated in the gray circle at genus level (or at the lowest taxonomic rank). Letters g, f, and o refer to the ranks genus, family and order, respectively. The outer circle specifies the phylum of each OTU.

It mainly concerns the *Betaproteobacteria* and the *Clostridia* (Schrenk et al., 2013). The abundance of *Betaproteobacteria* often exceeded 20% in the terrestrial systems studied herein (Daae et al., 2013; Quéméneur et al., 2015; Suzuki et al., 2013; Tiago and Veríssimo, 2013). Four *Betaproteobacteria* OTUs, found in both PHF sites and continental serpentinizing springs, were assigned to the H_2_-consuming genus *Hydrogenophaga* (**Figure 5**). Although not exclusive of serpentinizing systems, these OTUs were otherwise distributed in similar geothermal environments such as deep hydrothermal groundwater and sulphidic springs (Supplementary Table S5). This suggests that similar geochemical settings (presence of H_2_ rich fluids and oxic-anoxic interfaces) may have selected *Hydrogenophaga* species which metabolic capabilities are well adapted to these particular conditions. Indeed, 16S rRNA gene and, in some cases, related NiFe hydrogenases genes of *Hydrogenophaga* spp. were previously shown to be abundant in the serpentinizing-hosted systems listed in the comparison and others such as the springs of the Tablelands Ophiolite (Brazelton et al., 2012) or the Samail Ophiolite (Rempfert et al., 2017). It is noteworthy that no OTU representative of its sister genus *Serpentinomonas* was identified in PHF. These hyperalkaliphilic hydrogenotrophs isolated from The Cedars (Suzuki et al., 2014) have so far been detected only in terrestrial serpentinizing sites where they can be dominant, e.g., one OTU representing up to 14% of the sequences in Voltri (Quéméneur et al., 2015), or even constituting the unique OTU of *Betaproteobacteria*, accounting for 50% of the sequences, in the most alkaline well CSW1.1 (pH12.2) of CROMO but absent in the other wells with lower pH values (Twing et al., 2017). Otherwise, the submarine PHF sites shared a *Ralstonia* OTU (EU030486.1) with Lost-City, emphasizing the diversity of microorganisms that could oxidize hydrogen in the PHF sites.

In the *Clostridia* class, 4 OTUs representing clone sequences of PHF were also detected at CVA, at The Cedars. However, phylotypes closely related to these four OTUs were more generally distributed in other serpentinizing sites including Voltri and CROMO. As noticed for the *Hydrogenophaga* OTUs, these 4 OTUs were restricted to serpentinizing systems or to groundwater. The OTUs AM778006.1 and AB476673.1 were close to the candidate group SRB2 found in CROMO (Twing et al., 2017), while AM777965.1 was affiliated to the *Dethiobacter* genus, of which the only described species, *D. alkaliphilus*, is strictly anaerobic with a respiration metabolism and able to use acetate and sugars with sulfur, polysulfides, or thiosulfate as an electron acceptor (Sorokin et al., 2008). This bacterium has also been shown to be capable of chemolithoautotrophic growth by disproportion of elemental sulfur (a kind of mineral fermentation), a metabolic process which is more favorable under alkaline conditions (Poser et al., 2013) such those encountered in serpentinizing systems.

Regarding the archaeal community, the present comparison included the uncultivated *Methanosarcinales* lineages only detected in serpentinizing ecosystems (Quéméneur et al., 2014, 2015; Schrenk et al., 2004; Suzuki et al., 2013). The originality of PHF is provided by the simultaneous presence of the two *Methanosarcinales* OTUs: the LCMS and TCMS phylotypes (**Figure 5** and Supplementary Table S6).

#### OTUs Belonging to Dominant Taxa in PHF Not Generally Distributed in Other Sites

In the PHF, uncultured *Chloroflexi* and members of the class *Alphaproteobacteria* were the main taxa, discriminating the submarine sites from the intertidal one. The large *Chloroflexi* fraction of the PHF submarine sites was not commonly recovered among other serpentinizing systems, except in less active or extinct chimneys of the Lost-City Hydrothermal Field (Brazelton et al., 2010) and in deep ground water of The Cedars (Suzuki et al., 2013). Additionally, the ecosystem of The Cedars shared two uncultivated *Chloroflexi* OTU with PHF (**Figure 5**). The high-abundance level of *Alphaproteobacteria* (64.7%) in the intertidal BdJ site was also not representative of all serpentinizing systems, as exemplified by their low abundance in CVA and The Cedars ecosystems (Suzuki et al., 2013; Tiago and Veríssimo, 2013). The PHF sites shared, however, five *Alphaproteobacteria* OTUs assigned to N_2_-fixing *Rhizobiales*, chemoheterotrophic *Caulobacterales* and *Sphingomonadale*s with the Leka ophiolite or Voltri. *Thaumarchaeota* of the Marine Group I (MG-I) class (Supplementary Tables S4, S6) also represented a dominant taxon in half of the PHF sites as outlined above. One PHF OTU (AY505046.1) was also detected within a group of new MG-I sequences from Lost-City (Brazelton et al., 2006) (**Figure 5**). These sequences may represent a new ecotype of *Thaumarchaeota* specifically adapted to serpentinizing hydrothermal system conditions and, in particular, to low ammonium concentrations (Russell et al., 2010) and high pH values (>10). Precisely, pH adaptation has shown evidence to be the major driving force in *Thaumarchaeota* evolutionary diversification (Gubry-rangin et al., 2015). Thus, serpentinite-hosted systems may represent a niche for specialized alkaliphilic *Thaumarchaeota*.

#### OTUs Belonging to Alkaliphilic Cosmopolitan Taxa Shared with Other Serpentinizing Sites

Two *Gammaproteobacteri*a OTUs assigned to the genus *Silanimonas* (AM778014.1 and KF511881.1) were detected in BdJ and CVA and even in The Cedars and in Voltri for KF511881.1. The genus *Silanimonas* contains three species (Chun et al., 2017), two of which are slightly thermophilic alkaliphiles with pH optima for growth around 9–10. Although OTU AM778014.1 seems restricted to serpentinizing sites, OTU KF511881.1 is also commonly detected in various types of aquatic habitat such as lake water, marine sediment or hot springs. Considering the presence of *Silanimonas* in the four serpentinizing systems with a maximum pH around 11.5–12.5 and its absence in the Leka ophiolite (where the maximum in situ pH is 9.6; Daae et al., 2013), this genus might be an indicator of a highly alkaline environment rather than terrestrial serpentinizing ecosystems. A similar conclusion may be drawn for the *Trueper*a OTU (*Deinococcus-Thermus* phylum) shared by the PHF sites with high pH fluid, Voltri and CVA. Members of the genus *Trueper*a are known to be chemoorganotrophic alkaliphiles, slightly thermophilic and extremely resistant to ionizing radiation.

#### OTUs Endemic to the PHF Belonging to Candidate Phyla

The OTUs classified into the six bacterial candidate phyla detected in PHF were not recovered in other serpentinizing systems, with the exception of one *Parcubacteria* OTU (LN561460.1) identified in both the submarine ST07 site and the Voltri terrestrial springs. Members of this candidate phylum could be involved in ‘dark’ anaerobic fermentation (Castelle et al., 2017). Several metagenomic reconstructed genomes of *Parcubacteria* are very small with reduced metabolic capabilities (e.g., lacking ATP synthase genes), suggesting that they may be intercellular symbionts of other prokaryotes sharing the same habitat (Nelson and Stegen, 2015; Suzuki et al., 2017). Since some *Parcubacteria* members were found in abundance in the deep groundwater of The Cedars (as well as in Lost-City and in PHF) they were thought to be selected by the geochemical conditions associated with serpentinization reactions (Suzuki et al., 2017). However, no *Parcubacteria* OTU was shared by PHF and The Cedars (**Figure 5**) and their closest OTUs indicated less than 90% identity on their overlapping region. Although clone sequencing of The Cedars (Suzuki et al., 2013) may have failed to identify the overall *Parcubacteria* community, PHF most likely hosted distantly related taxa potentially associated with other metabolisms and different microbial consortia.

## Conclusion

In this study, 16S rRNA gene pyrosequencing provided a comprehensive overview of the microbial biosphere of PHF and extended its known diversity by revealing the presence of prokaryotic populations pertaining to six novel phyla not previously detected. Moreover, this study showed that bacterial and archaeal communities in PHF were dominated by rare phylotypes which, for the most part, appeared to be endemic to the PHF ecosystem. This study also included the first exhaustive comparison at OTU scale of microbial taxa across six geographically distant serpentinite-hosted ecosystems. Although at this taxonomic resolution, no stricto sensu core microbial community could be defined, a few phylotypes were shared by several sites. These phylotypes, belonging to generally dominant taxa in serpentinizing systems, seem to be specifically associated with unique niches resulting from the peculiar geochemical conditions linked to serpentinization. Indeed, OTUs representing uncultivated *Clostridia* of the *Syntrophomonaceae* family (and related to the *Dethiobacter alkaliphilus*) or of the *Thermoanaerobacterales* group SRB2 are hypothesized to live in the anoxic, H_2_ rich end-member fluids or inside the porous carbonates structures, alike the *Methanosarcinales* (LCMS and TCMS) (Schrenk et al., 2013). *Hydrogenophaga* OTUs identified in most of the terrestrial serpentinizing sites probably live at oxic-anoxic interfaces where the mixing of alkaline H_2_-rich fluids and oxygenated waters creates microaerophilic conditions. Upon mixing, steep gradients of pH, temperature and electron donors and acceptors, also provide a range of microhabitats favorable for endemic microbial populations in these extreme ecosystems. Additional factors, abiotic (geological and hydrological settings, water depths, light, etc.) or biotic (species interactions, viruses), superimposed to the serpentinization reactions most likely contributed significantly in shaping the microbial assemblages, thus explaining the limited similarities between ecosystems at the OTU level. This apparent divergence of microbiome composition in serpentinizing systems raised the question of the existence of common metabolic capabilities that could explain the functioning of these ecosystems better. These issues could be addressed in the future by using new genomic tools, such as comparative metagenomics.

## Author Contributions

GE designed the study. AP, GE, and MQ performed the field sampling. The experiments were carried out by MB and GE. EF, MB, and FA performed the bioinformatics analyses and interpretations. EF, MB, FA, and GE wrote the manuscript. AP, MQ, BO, and DD reviewed drafts of the paper.

## Conflict of Interest Statement

The authors declare that the research was conducted in the absence of any commercial or financial relationships that could be construed as a potential conflict of interest.
