# Supplementary material for: Diversity of Rare and Abundant Prokaryotic Phylotypes in the Prony Hydrothermal Field and Comparison with Other Serpentinite-Hosted Ecosystems
Source: Front Microbiol. 2018 Feb 6;9:102. doi: 10.3389/fmicb.2018.00102 (PMC5808123; doi:10.3389/fmicb.2018.00102)
Supplement: Supplementary file 1 [file Table_1.DOCX]

**Supplementary Table 1.** Samples and multiplex identifiers.

| **Site** | **Sampling date** | **MID** | **Sequence** |
| --- | --- | --- | --- |
| ST09 | 2010 | MID1 | ACG-AGT-GCG-T |
| ST07 | 2012 | MID2 | ACG-CTC-GAC-A |
| ST12 | 2011 | MID3 | AGA-CGC-ACT-C |
| BdJ | 2011 | MID4 | AGC-ACT-GTA-G |
