# Supplementary material for: Diversity of Rare and Abundant Prokaryotic Phylotypes in the Prony Hydrothermal Field and Comparison with Other Serpentinite-Hosted Ecosystems
Source: Front Microbiol. 2018 Feb 6;9:102. doi: 10.3389/fmicb.2018.00102 (PMC5808123; doi:10.3389/fmicb.2018.00102)
Supplement: Supplementary file 2 [file Table_2.DOCX]

Supplementary Table 2. Basic statistics of OTUs distribution and diversity indexes.

|  | | **Archaea** | | | | **Bacteria** | | | |
| --- | --- | --- | --- | --- | --- | --- | --- | --- | --- |
|  |  | BdJ | ST07 | ST09 | ST12 | BdJ | ST07 | ST09 | ST12 |
| **Trimmed sequences** | | 21395 | 26167 | 20958 | 27396 | 15090 | 15431 | 15731 | 28205 |
| **Number of OTUs** | | **47** | **213** | **242** | **132** | **382** | **626** | **583** | **1203** |
| **Diversity indexes** | Shannon | 1.72 | 3.04 | 3.76 | 1.88 | 4.19 | 5.03 | 4.66 | 4.98 |
|  | Simpson | 0.76 | 0.88 | 0.96 | 0.74 | 0.95 | 0.98 | 0.96 | 0.92 |
| **Rare OTUs (<0.2 %)** | number of OTUs | **34** | **168** | **190** | **115** | **324** | **548** | **513** | **1146** |
|  | % rare OTUs | 72.3 | 78.9 | 78.5 | 87.1 | 84.8 | 87.5 | 88.0 | 95.3 |
|  | % of sequences | 1.7 | 7.4 | 8.8 | 4.6 | 20.3 | 28.0 | 26.8 | 42.8 |
| **Intermediate OTUs (0.2-1%)** | number of OTUs | **7** | **34** | **30** | **12** | **42** | **59** | **56** | **54** |
|  | % interm. OTUs | 14.9 | 16.0 | 12.4 | 9.1 | 11.0 | 9.4 | 9.6 | 4.5 |
|  | % of sequences | 2.7 | 16.9 | 12.7 | 5.3 | 18.8 | 24.8 | 23.3 | 19.4 |
| **Abundant OTUs (>1 %)** | number of OTUs | **6** | **11** | **22** | **5** | **16** | **19** | **14** | **3** |
|  | % abundant OTUs | 12.8 | 5.2 | 9.1 | 3.8 | 4.2 | 3.0 | 2.4 | 0.2 |
|  | % of sequences | 95.5 | 75.7 | 78.5 | 90.1 | 60.9 | 47.2 | 50.0 | 37.7 |
