# Supplementary material for: Diversity of Rare and Abundant Prokaryotic Phylotypes in the Prony Hydrothermal Field and Comparison with Other Serpentinite-Hosted Ecosystems
Source: Front Microbiol. 2018 Feb 6;9:102. doi: 10.3389/fmicb.2018.00102 (PMC5808123; doi:10.3389/fmicb.2018.00102)
Supplement: Supplementary file 3 [file Table_3.DOCX]

Supplementary Table 3. Taxonomy details of abundant bacterial OTUs. The representative sequences of each OTU were blasted against RefSeq database to identify their closest relatives.

| OTU ID | ST12 | BdJ | ST07 | ST09 | Taxonomy | Closest relatives retrieved from RefSeq database | | |
| --- | --- | --- | --- | --- | --- | --- | --- | --- |
|  |  |  |  |  |  | Bacterial strain | Genbank ID | % identity |
| OTU149 | 17 | 0 | 62 | 249 | k_Bacteria; p_Acidobacteria; c_Acidobacteria; o_Subgroup 11; f_uncultured Acidobacteria bacterium;  g_uncultured Acidobacteria bacterium; s_uncultured Acidobacteria bacterium | Silvibacterium bohemicum | NR_135209.1 | 82% |
| OTU167 | 6 | 0 | 33 | 391 | k_Bacteria; p_Actinobacteria; c_Acidimicrobiia; o_Acidimicrobiales; f_Sva0996 marine group;  g_uncultured Acidimicrobiales bacterium; s_uncultured Acidimicrobiales bacterium | Aciditerrimonas ferrireducens JCM 15389 | NR_112972.1 | 90% |
| OTU1388 | 4 | 0 | 0 | 140 | k_Bacteria; p_Bacteroidetes; c_Sphingobacteriia; o_Sphingobacteriales | Crenotalea thermophila | NR_125473.1 | 82% |
| OTU753 | 0 | 0 | 402 | 0 | k_Bacteria; p_Chloroflexi; c_Anaerolineae; o_Anaerolineales; f_Anaerolineaceae;  g_uncultured; s_uncultured Chloroflexus sp. | Saccharomonospora viridis | NR_074713.2 | 81% |
| OTU793 | 0 | 0 | 175 | 9 | k_Bacteria; p_Chloroflexi; c_Anaerolineae; o_Anaerolineales; f_Anaerolineaceae;  g_uncultured; s_uncultured Chloroflexus sp. | Nocardia camponoti | NR_148835.1 | 83% |
| OTU1317 | 0 | 0 | 181 | 0 | k_Bacteria; p_Chloroflexi; c_Anaerolineae; o_Anaerolineales; f_Anaerolineaceae;  g_uncultured; s_uncultured Chloroflexus sp. | Ornatilinea apprima | NR_109544.1 | 80% |
| OTU1604 | 0 | 0 | 162 | 0 | k_Bacteria; p_Chloroflexi; c_Anaerolineae; o_Anaerolineales; f_Anaerolineaceae;  g_uncultured; s_uncultured Chloroflexus sp. | Saccharomonospora viridis | NR_074713.2 | 81% |
| OTU641 | 11 | 0 | 177 | 153 | k_Bacteria; p_Chloroflexi; c_Caldilineae; o_Caldilineales; f_Caldilineaceae;  g_uncultured; s_uncultured Chloroflexus sp. | Litorilinea aerophila | NR_132330.1 | 84% |
| OTU1432 | 25 | 0 | 682 | 5 | k_Bacteria; p_Chloroflexi; c_Dehalococcoidia; o_GIF9 | Dehalogenimonas lykanthroporepellens | NR_074337.1 | 81% |
| OTU1706 | 891 | 0 | 345 | 25 | k_Bacteria; p_Chloroflexi; c_Dehalococcoidia; o_GIF9 | Dehalogenimonas lykanthroporepellens | NR_074337.1 | 81% |
| OTU1947 | 8 | 0 | 557 | 23 | k_Bacteria; p_Chloroflexi; c_Dehalococcoidia; o_GIF9 | Dehalogenimonas alkenigignens | NR_109657.1 | 82% |
| OTU1117 | 2915 | 0 | 504 | 1122 | k_Bacteria; p_Chloroflexi; c_Dehalococcoidia; o_MSBL5 | Lihuaxuella thermophila | NR_126245.2 | 80% |
| OTU679 | 295 | 0 | 14 | 27 | k_Bacteria; p_Chloroflexi; c_Dehalococcoidia; o_MSBL5; f_uncultured bacterium;  g_uncultured bacterium; s_uncultured bacterium | Acidimicrobium ferrooxidans | NR_074390.1 | 80% |
| OTU915 | 15 | 0 | 442 | 1346 | k_Bacteria; p_Chloroflexi; c_Dehalococcoidia; o_MSBL5; f_uncultured bacterium;  g_uncultured bacterium; s_uncultured bacterium | Acidimicrobium ferrooxidans | NR_074390.1 | 81% |
| OTU1039 | 25 | 1 | 225 | 696 | k_Bacteria; p_Chloroflexi; c_Dehalococcoidia; o_MSBL5; f_uncultured bacterium;  g_uncultured bacterium; s_uncultured bacterium | Acidimicrobium ferrooxidans | NR_074390.1 | 81% |
| OTU1050 | 0 | 0 | 28 | 170 | k_Bacteria; p_Chloroflexi; c_Dehalococcoidia; o_MSBL5; f_uncultured bacterium;  g_uncultured bacterium; s_uncultured bacterium | Lihuaxuella thermophila | NR_126245.2 | 81% |
| OTU1275 | 77 | 0 | 99 | 135 | k_Bacteria; p_Chloroflexi; c_Dehalococcoidia; o_MSBL5; f_uncultured bacterium;  g_uncultured bacterium; s_uncultured bacterium | Acidimicrobium ferrooxidans | NR_074390.1 | 82% |
| OTU1529 | 5 | 0 | 55 | 222 | k_Bacteria; p_Chloroflexi; c_Dehalococcoidia; o_MSBL5; f_uncultured bacterium;  g_uncultured bacterium; s_uncultured bacterium | Lihuaxuella thermophila | NR_126245.2 | 81% |
| OTU161 | 35 | 0 | 67 | 155 | k_Bacteria; p_Chloroflexi; c_SAR202 clade | Roseiflexus castenholzii | NR_074188.1 | 81% |
| OTU300 | 32 | 0 | 333 | 14 | k_Bacteria; p_Chloroflexi; c_SAR202 clade | Roseiflexus castenholzii | NR_074188.1 | 81% |
| OTU1534 | 0 | 153 | 0 | 0 | k_Bacteria; p_Cyanobacteria; c_Cyanobacteria; o_SubsectionIII; f_FamilyI | Halomicronema excentricum | NR_114591.1 | 90% |
| OTU1127 | 0 | 150 | 0 | 0 | k_Bacteria; p_Cyanobacteria; c_Cyanobacteria; o_SubsectionIII; f_FamilyI;  g_Phormidium | Synechococcus elongatus | NR_074309.1 | 91% |
| OTU968 | 0 | 120 | 0 | 0 | k_Bacteria; p_Cyanobacteria; c_Cyanobacteria; o_SubsectionIII; f_FamilyI;  g_Prochlorothrix; s_uncultured marine bacterium | Microcystis aeruginosa | NR_074314.1 | 92% |
| OTU1474 | 0 | 252 | 0 | 0 | k_Bacteria; p_Cyanobacteria; c_Cyanobacteria; o_SubsectionIII; f_FamilyI;  g_Prochlorothrix; s_uncultured marine bacterium | Microcystis aeruginosa | NR_074314.1 | 92% |
| OTU1266 | 0 | 151 | 0 | 0 | k_Bacteria; p_Deinococcus-Thermus; c_Deinococci; o_Thermales; f_Thermaceae;  g_Meiothermus; s_uncultured bacterium | Meiothermus hypogaeus NBRC 106114 | NR_113226.1 | 95% |
| OTU796 | 9 | 0 | 217 | 411 | k_Bacteria; p_Firmicutes; c_Clostridia; o_Clostridiales; f_Syntrophomonadaceae;  g_uncultured; s_uncultured Firmicutes bacterium | Natranaerobaculum magadiense | NR_135713.1 | 84% |
| OTU135 | 3 | 0 | 144 | 18 | k_Bacteria; p_Gemmatimonadetes; c_Gemmatimonadetes; o_PAUC43f marine benthic group; f_uncultured sponge symbiont PAUC51f; g_uncultured sponge symbiont PAUC51f; s_uncultured sponge symbiont PAUC51f | Eilatimonas milleporae | NR_109076.1 | 82% |
| OTU234 | 16 | 0 | 115 | 115 | k_Bacteria; p_Gemmatimonadetes; c_Gemmatimonadetes; o_PAUC43f marine benthic group; f_uncultured sponge symbiont PAUC51f; g_uncultured sponge symbiont PAUC51f; s_uncultured sponge symbiont PAUC51f | Eilatimonas milleporae | NR_109076.1 | 81% |
| OTU266 | 3 | 2 | 118 | 76 | k_Bacteria; p_Proteobacteria; c_Alphaproteobacteria; o_Rhizobiales; f_Rhodobiaceae;  g_Methyloceanibacter; s_uncultured bacterium | Methyloceanibacter caenitepidi | NR_125465.1 | 98% |
| OTU594 | 1 | 1 | 113 | 61 | k_Bacteria; p_Proteobacteria; c_Alphaproteobacteria; o_Rhizobiales; f_Rhodobiaceae;  g_Methyloceanibacter; s_uncultured bacterium | Methyloceanibacter caenitepidi | NR_125465.1 | 98% |
| OTU680 | 0 | 114 | 0 | 0 | k_Bacteria; p_Proteobacteria; c_Alphaproteobacteria; o_Rhodobacterales; f_Rhodobacteraceae | Pseudooceanicola antarcticus | NR_134107.1 | 96% |
| OTU1285 | 0 | 271 | 0 | 0 | k_Bacteria; p_Proteobacteria; c_Alphaproteobacteria; o_Rhodobacterales; f_Rhodobacteraceae | Paracoccus siganidrum | NR_118463.1 | 96% |
| OTU1946 | 0 | 217 | 0 | 0 | k_Bacteria; p_Proteobacteria; c_Alphaproteobacteria; o_Rhodobacterales; f_Rhodobacteraceae | Loktanella litorea | NR_118329.1 | 99% |
| OTU279 | 30 | 1340 | 38 | 16 | k_Bacteria; p_Proteobacteria; c_Alphaproteobacteria; o_Rhodobacterales; f_Rhodobacteraceae;  g_Roseibaca | Roseinatronobacter monicus | NR_043914.1 | 96% |
| OTU1806 | 4 | 1037 | 6 | 8 | k_Bacteria; p_Proteobacteria; c_Alphaproteobacteria; o_Rhodobacterales; f_Rhodobacteraceae;  g_Roseibaca | Paenirhodobacter enshiensis | NR_125604.1 | 95% |
| OTU153 | 4 | 1239 | 29 | 0 | k_Bacteria; p_Proteobacteria; c_Alphaproteobacteria; o_Rhodobacterales; f_Rhodobacteraceae;  g_Roseibaca; s_uncultured bacterium | Roseinatronobacter monicus | NR_043914.1 | 96% |
| OTU1298 | 2 | 417 | 2 | 0 | k_Bacteria; p_Proteobacteria; c_Alphaproteobacteria; o_Rhodobacterales; f_Rhodobacteraceae;  g_Roseibaca; s_uncultured bacterium | Roseinatronobacter monicus | NR_043914.1 | 96% |
| OTU1067 | 0 | 122 | 0 | 0 | k_Bacteria; p_Proteobacteria; c_Alphaproteobacteria; o_Rhodobacterales; f_Rhodobacteraceae; g_Tropicimonas; s_uncultured bacterium | Dinoroseobacter shibae | NR_074166.1 | 95% |
| OTU137 | 0 | 0 | 114 | 0 | k_Bacteria; p_Proteobacteria; c_Alphaproteobacteria; o_Rickettsiales; f_SAR116 clade | Nisaea nitritireducens | NR_043924.1 | 91% |
| OTU429 | 0 | 0 | 122 | 0 | k_Bacteria; p_Proteobacteria; c_Alphaproteobacteria; o_Rickettsiales; f_SAR116 clade | Nisaea nitritireducens | NR_043924.1 | 91% |
| OTU607 | 7 | 654 | 0 | 0 | k_Bacteria; p_Proteobacteria; c_Betaproteobacteria; o_Burkholderiales; f_Comamonadaceae | Hydrogenophaga defluvii | NR_029024.1 | 98% |
| OTU763 | 4 | 230 | 0 | 0 | k_Bacteria; p_Proteobacteria; c_Betaproteobacteria; o_Burkholderiales; f_Comamonadaceae | Hydrogenophaga defluvii | NR_029024.1 | 98% |
| OTU933 | 0 | 0 | 1 | 123 | k_Bacteria; p_Proteobacteria; c_Deltaproteobacteria; o_Bdellovibrionales; f_Bdellovibrionaceae; g_Bdellovibrio | Bdellovibrio exovorus | NR_102876.1 | 81% |
| OTU1347 | 10 | 155 | 15 | 26 | k_Bacteria; p_Proteobacteria; c_Deltaproteobacteria; o_Desulfovibrionales; f_Desulfonatronaceae; g_Desulfonatronum; s_uncultured bacterium | Desulfonatronum alkalitolerans | NR_108631.1 | 97% |
