# Supplementary material for: Diversity of Rare and Abundant Prokaryotic Phylotypes in the Prony Hydrothermal Field and Comparison with Other Serpentinite-Hosted Ecosystems
Source: Front Microbiol. 2018 Feb 6;9:102. doi: 10.3389/fmicb.2018.00102 (PMC5808123; doi:10.3389/fmicb.2018.00102)
Supplement: Supplementary file 4 [file Table_4.DOCX]

Supplementary Table 4. Taxonomy details of abundant archaeal OTUs. The affiliations at the genus level of OTU155, OTU192, OTU294, OTU174, OTU280, OTU303, OTU16, OTU184, OTU193 were automatically generated by the RDP pipeline. They slightly differed from the phylogenetic annotations presented in Figure 4 due to some discrepancies of the *Methanosarcinales* classification in the Silva database. The representative sequences of each OTU were blasted against RefSeq database to identify their closest relatives.

| OTU ID | ST09 | ST07 | ST12 | BdJ | Taxonomy | Closest relatives retrieved from RefSeq database | | |
| --- | --- | --- | --- | --- | --- | --- | --- | --- |
|  |  |  |  |  |  | Archaeal strain | Genbank ID | % identity |
| OTU155 | 805 | 32 | 797 | 2467 | k__Archaea; p__Euryarchaeota; c__Methanomicrobia; o__Methanosarcinales; f__Methanosarcinaceae | Methanolobus psychrophilus R15 | NR_102921.1 | 92% |
| OTU192 | 0 | 0 | 141 | 257 | k__Archaea; p__Euryarchaeota; c__Methanomicrobia; o__Methanosarcinales; f__Methanosarcinaceae | Methanolobus psychrophilus R15 | NR_115986.1 | 90% |
| OTU294 | 2237 | 78 | 74 | 68 | k__Archaea; p__Euryarchaeota; c__Methanomicrobia; o__Methanosarcinales; f__Methanosarcinaceae; g__ANME-3 | Methanolobus psychrophilus R15 | NR_115986.1 | 92% |
| OTU174 | 1105 | 42 | 7080 | 7281 | k__Archaea; p__Euryarchaeota; c__Methanomicrobia; o__Methanosarcinales; f__Methanosarcinaceae; g__ANME-3; s__uncultured archaeon | Methanolobus psychrophilus R15 | NR_102921.1 | 91% |
| OTU280 | 0 | 0 | 6609 | 4652 | k__Archaea; p__Euryarchaeota; c__Methanomicrobia; o__Methanosarcinales; f__Methanosarcinaceae; g__ANME-3; s__uncultured archaeon | Methanolobus psychrophilus R15 | NR_115986.1 | 92% |
| OTU303 | 634 | 17 | 1501 | 3288 | k__Archaea; p__Euryarchaeota; c__Methanomicrobia; o__Methanosarcinales; f__Methanosarcinaceae; g__ANME-3; s__uncultured archaeon | Methanomethylovorans hollandica DSM 15978 | NR_102454.1 | 91% |
| OTU16 | 1428 | 4926 | 1565 | 662 | k__Archaea; p__Euryarchaeota; c__Methanomicrobia; o__Methanosarcinales; f__Methermicoccaceae; g__Methermicoccus; s__uncultured bacterium | Methanoculleus sediminis | NR_136474.1 | 88% |
| OTU184 | 86 | 266 | 77 | 45 | k__Archaea; p__Euryarchaeota; c__Methanomicrobia; o__Methanosarcinales; f__Methermicoccaceae; g__Methermicoccus; s__uncultured bacterium | Methanoculleus sediminis | NR_136474.1 | 88% |
| OTU193 | 153 | 389 | 115 | 102 | k__Archaea; p__Euryarchaeota; c__Methanomicrobia; o__Methanosarcinales; f__Methermicoccaceae; g__Methermicoccus; s__uncultured bacterium | Methanosaeta harundinacea | NR_043203.1 | 87% |
| OTU20 | 238 | 93 | 2 | 0 | k__Archaea; p__Thaumarchaeota; c__Marine Group I | Nitrosopumilus maritimus SCM1 | NR_102913.1 | 92% |
| OTU61 | 388 | 169 | 4 | 0 | k__Archaea; p__Thaumarchaeota; c__Marine Group I | Nitrosopumilus maritimus SCM1 | NR_102913.1 | 92% |
| OTU78 | 268 | 87 | 4 | 0 | k__Archaea; p__Thaumarchaeota; c__Marine Group I | Nitrosopumilus maritimus SCM1 | NR_102913.1 | 92% |
| OTU2 | 228 | 157 | 34 | 0 | k__Archaea; p__Thaumarchaeota; c__Marine Group I; o__Unknown Order; f__Unknown Family; g__Candidatus Nitrosopumilus | Nitrosopumilus maritimus SCM1 | NR_102913.1 | 98% |
| OTU35 | 232 | 152 | 39 | 0 | k__Archaea; p__Thaumarchaeota; c__Marine Group I; o__Unknown Order; f__Unknown Family; g__Candidatus Nitrosopumilus | Nitrosopumilus maritimus SCM1 | NR_102913.1 | 98% |
| OTU56 | 432 | 254 | 15 | 0 | k__Archaea; p__Thaumarchaeota; c__Marine Group I; o__Unknown Order; f__Unknown Family; g__Candidatus Nitrosopumilus | Nitrosopumilus maritimus SCM1 | NR_102913.1 | 97% |
| OTU109 | 1070 | 594 | 119 | 0 | k__Archaea; p__Thaumarchaeota; c__Marine Group I; o__Unknown Order; f__Unknown Family; g__Candidatus Nitrosopumilus | Nitrosopumilus maritimus SCM1 | NR_102913.1 | 98% |
| OTU114 | 444 | 445 | 61 | 0 | k__Archaea; p__Thaumarchaeota; c__Marine Group I; o__Unknown Order; f__Unknown Family; g__Candidatus Nitrosopumilus | Nitrosopumilus maritimus SCM1 | NR_102913.1 | 98% |
| OTU128 | 245 | 86 | 35 | 0 | k__Archaea; p__Thaumarchaeota; c__Marine Group I; o__Unknown Order; f__Unknown Family; g__Candidatus Nitrosopumilus | Nitrosopumilus maritimus SCM1 | NR_102913.1 | 98% |
| OTU149 | 239 | 133 | 94 | 0 | k__Archaea; p__Thaumarchaeota; c__Marine Group I; o__Unknown Order; f__Unknown Family; g__Candidatus Nitrosopumilus | Nitrosopumilus maritimus SCM1 | NR_102913.1 | 99% |
| OTU172 | 270 | 103 | 8 | 0 | k__Archaea; p__Thaumarchaeota; c__Marine Group I; o__Unknown Order; f__Unknown Family; g__Candidatus Nitrosopumilus | Nitrosopumilus maritimus SCM1 | NR_102913.1 | 95% |
| OTU188 | 124 | 335 | 13 | 0 | k__Archaea; p__Thaumarchaeota; c__Marine Group I; o__Unknown Order; f__Unknown Family; g__Candidatus Nitrosopumilus | Nitrosopumilus maritimus SCM1 | NR_102913.1 | 97% |
| OTU198 | 235 | 48 | 8 | 0 | k__Archaea; p__Thaumarchaeota; c__Marine Group I; o__Unknown Order; f__Unknown Family; g__Candidatus Nitrosopumilus | Nitrosopumilus maritimus SCM1 | NR_102913.1 | 98% |
| OTU31 | 1103 | 3746 | 79 | 0 | k__Archaea; p__Thaumarchaeota; c__Marine Group I; o__Unknown Order; f__Unknown Family; g__Candidatus Nitrosopumilus; s__uncultured Nitrosopumilales archaeon | Nitrosopumilus maritimus SCM1 | NR_102913.1 | 96% |
| OTU86 | 817 | 1388 | 37 | 0 | k__Archaea; p__Thaumarchaeota; c__Marine Group I; o__Unknown Order; f__Unknown Family; g__Candidatus Nitrosopumilus; s__uncultured Nitrosopumilales archaeon | Nitrosopumilus maritimus SCM1 | NR_102913.1 | 96% |
| OTU113 | 684 | 2056 | 32 | 0 | k__Archaea; p__Thaumarchaeota; c__Marine Group I; o__Unknown Order; f__Unknown Family; g__Candidatus Nitrosopumilus; s__uncultured Nitrosopumilales archaeon | Nitrosopumilus maritimus SCM1 | NR_102913.1 | 96% |
| OTU146 | 121 | 351 | 14 | 0 | k__Archaea; p__Thaumarchaeota; c__Marine Group I; o__Unknown Order; f__Unknown Family; g__Candidatus Nitrosopumilus; s__uncultured Nitrosopumilales archaeon | Nitrosopumilus maritimus SCM1 | NR_102913.1 | 97% |
| OTU194 | 1244 | 146 | 27 | 0 | k__Archaea; p__Thaumarchaeota; c__Marine Group I; o__Unknown Order; f__Unknown Family; g__Candidatus Nitrosopumilus; s__uncultured Nitrosopumilales archaeon | Nitrosopumilus maritimus SCM1 | NR_102913.1 | 95% |
| OTU272 | 945 | 165 | 23 | 0 | k__Archaea; p__Thaumarchaeota; c__Marine Group I; o__Unknown Order; f__Unknown Family; g__Candidatus Nitrosopumilus; s__uncultured Nitrosopumilales archaeon | Nitrosopumilus maritimus SCM1 | NR_102913.1 | 95% |
