# Supplementary material for: Diversity of Rare and Abundant Prokaryotic Phylotypes in the Prony Hydrothermal Field and Comparison with Other Serpentinite-Hosted Ecosystems
Source: Front Microbiol. 2018 Feb 6;9:102. doi: 10.3389/fmicb.2018.00102 (PMC5808123; doi:10.3389/fmicb.2018.00102)
Supplement: Supplementary file 5 [file Table_5.DOCX]

Supplementary Table 5. Taxonomy details of bacterial OTUs shared at least by one sample from PHF (this study, Pisapia et al., 2017; Postec et al., 2015; Quéméneur et al., 2014) and by one sample from another serpentinizing system among Cabeço de Vide Aquifer (Tiago and Veríssimo, 2013), The Cedars (Suzuki et al., 2013), Voltri ophiolite (Quéméneur et al., 2015), Leka ophiolite (Daae et al., 2013) and Lost-City (Brazelton et al., 2006; Schrenk et al., 2004). If the representative sequence of an OTU was isolated in a serpentinizing ecosystem, the last two columns indicate the closest relative of this sequence, identified in another kind of environment (i.e. not influenced by serpentinization reaction).

| **OTUs and their distribution in studied serpentinizing systems** | | | | | **Closest relative in ecosystems that are not reported as serpentinizing sites** | |
| --- | --- | --- | --- | --- | --- | --- |
| **Genbank ID ^(*)^** | **Taxonomy (p_Phylum; c_Class; o_Order; f_Family; g_Genus; s_Species)** | **PHF sites** | **Other ecosystems** | **Representative sequence source** | **Genbank ID (coverage, identity)** | **Environment** |
| AY741058.1 | p_Actinobacteria; c_Actinobacteria; o_Propionibacteriales; f_Propionibacteriaceae; g_Propionibacterium; s_Propionibacterium acnes | ST12 | CVA, Lost-City | Pericardial fluid |  |  |
| AM777981.1 | p_Actinobacteria; c_Coriobacteriia; o_Coriobacteriales; f_Coriobacteriaceae;  g_uncultured; s_uncultured bacterium | ST07 | CVA | Serpentinizing site | AB936639.1  (97%, 95%) | River sediment |
| GU056099.1 | p_Actinobacteria; c_Thermoleophilia; o_Solirubrobacterales; f_480-2;  g_uncultured bacterium; s_uncultured bacterium | BdJ | Voltri | Gas and oil field |  |  |
| KC574890.1 | p_Chloroflexi; c_Dehalococcoidia; o_MSBL5; f_uncultured bacterium;  g_uncultured bacterium; s_uncultured bacterium | ST12, ST09, ST08, ST07 | The Cedars | Serpentinizing site | JN207189.1  (100%, 92%) | Sediment of Namako-ike lake |
| JN123498.1 | p_Chloroflexi; c_Dehalococcoidia; o_vadinBA26; f_uncultured bacterium;  g_uncultured bacterium; s_uncultured bacterium | ST12 | The Cedars | Seafloor sediment |  |  |
| HQ433568.1 | p_Cyanobacteria; c_Chloroplast; o_uncultured bacterium; f_uncultured bacterium;  g_uncultured bacterium; s_uncultured bacterium | ST12 | Voltri | Shrimp pond |  |  |
| AB757744.1 | p_Cyanobacteria; c_Cyanobacteria; o_SubsectionI; f_FamilyI;  g_Synechococcus; s_uncultured bacterium | BdJ | The Cedars | Hot Spring Water |  |  |
| KF912961.1 | p_Deinococcus-Thermus; c_Deinococci; o_Deinococcales; f_Trueperaceae;  g_Truepera; s_uncultured bacterium | ST09, ST07, BdJ | Voltri, CVA | Chromium  soil contaminated |  |  |
| AM777965.1 | p_Firmicutes; c_Clostridia; o_Clostridiales; f_Syntrophomonadaceae;  g_Dethiobacter; s_uncultured bacterium | ST09 | CVA | Serpentinizing site | DQ088769.1  (93%, 98%) | Deep gold mine |
| DQ088764.1 | p_Firmicutes; c_Clostridia; o_D8A-2; f_uncultured bacterium;  g_uncultured bacterium; s_uncultured bacterium | ST12, ST09, ST08, ST07 | CVA | Deep gold mine |  |  |
| AM778006.1^(1)^ | p_Firmicutes; c_Clostridia; o_Thermoanaerobacterales; f_SRB2;  g_uncultured bacterium; s_uncultured bacterium | ST11 | CVA, The Cedars | Serpentinizing site | AB476673.1  (97%, 98%) | Sulfur containing freshwater source |
| AB476673.1 | p_Firmicutes; c_Clostridia; o_Thermoanaerobacterales; f_SRB2;  g_uncultured Firmicutes bacterium; s_uncultured Firmicutes bacterium | ST07 | CVA | Sulfur containing freshwater source |  |  |
| LN561460.1 | p_Parcubacteria; c_uncultured bacterium; o_uncultured bacterium; f_uncultured bacterium; g_uncultured bacterium; s_uncultured bacterium | ST07 | Voltri | Refuse dump |  |  |
| FJ612223.1 | p_Proteobacteria; c_Alphaproteobacteria; o_Caulobacterales; f_Hyphomonadaceae;  g_Hyphomonas; s_uncultured bacterium | BdJ | Voltri | Lake water |  |  |
| DQ125521.1 | p_Proteobacteria; c_Alphaproteobacteria; o_Rhizobiales; f_Hyphomicrobiaceae;  g_uncultured; s_uncultured bacterium | ST07 | Voltri | Uranium contaminated soil |  |  |
| JF119689.1 | p_Proteobacteria; c_Alphaproteobacteria; o_Rhizobiales; f_Hyphomicrobiaceae; g_Hyphomicrobium; s_uncultured bacterium | ST09 | Voltri | Skin |  |  |
| KF182250.1 | p_Proteobacteria; c_Alphaproteobacteria; o_Rhizobiales; f_Bradyrhizobiaceae;  g_Bradyrhizobium; s_uncultured bacterium | ST12 | Leka | Coal gangue dump soil |  |  |
| JN038233.1 | p_Proteobacteria; c_Alphaproteobacteria; o_Sphingomonadales; f_Erythrobacteraceae; g_Porphyrobacter; s_uncultured Sphingomonadales bacterium | BdJ | Voltri | Petroleum contaminated soil |  |  |
| JX521390.1 | p_Proteobacteria; c_Betaproteobacteria; o_Burkholderiales; f_Comamonadaceae; g_Hydrogenophaga; s_uncultured bacterium | BdJ | The Cedars | Terrestrial sulfidic spring |  |  |
| AF385534.1 | p_Proteobacteria; c_Betaproteobacteria; o_Burkholderiales; f_Comamonadaceae;  g_Schlegelella; s_Leptothrix sp. oral clone AW043 | ST09, ST07 | CVA | Noma lesions |  |  |
| KF300927.1 | p_Proteobacteria; c_Betaproteobacteria; o_Burkholderiales; f_Comamonadaceae; g_Hydrogenophaga; s_uncultured bacterium | BdJ | Voltri, Leka | Yellow River sediment |  |  |
| DQ256326.1 | p_Proteobacteria; c_Betaproteobacteria; o_Burkholderiales; f_Comamonadaceae; g_Hydrogenophaga; s_uncultured bacterium | ST07 | CVA, The Cedars | Subsurface water of the Kalahari Shield |  |  |
| KF441648.1 | p_Proteobacteria; c_Betaproteobacteria; o_Burkholderiales; f_Comamonadaceae; g_Hydrogenophaga; s_Hydrogenophaga sp. 7A-385 | ST12, ST07, BdJ | Leka | Urgeirica mine |  |  |
| EU030486.1 | p_Proteobacteria; c_Betaproteobacteria; o_Burkholderiales; f_Burkholderiaceae; g_Ralstonias_uncultured bacterium | ST12, ST09 | Lost-City | Antarctic ice sheet |  |  |
| KF733685.1 | p_Proteobacteria; c_Betaproteobacteria; o_Burkholderiales; f_Burkholderiaceae;  g_Burkholderia; s_Burkholderia ambifaria | ST12, ST07 | CVA | Oral cavity |  |  |
| EU470514.1 | p_Proteobacteria; c_Gammaproteobacteria; o_B38; f_uncultured bacterium;  g_uncultured bacterium; s_uncultured bacterium | ST12 | CVA | Red panda feces |  |  |
| KJ127981.1 | p_Proteobacteria; c_Gammaproteobacteria; o_Chromatiales; f_Chromatiaceae;  g_Nitrosococcus; s_uncultured Ectothiorhodospiraceae bacterium | BdJ | Voltri | Slime from paper machine |  |  |
| HQ739644.1 | p_Proteobacteria; c_Gammaproteobacteria; o_Enterobacteriales; f_Enterobacteriaceae; g_Escherichia-Shigella; s_uncultured bacterium | ST12 | CVA | Gastrointestinal specimens |  |  |
| KF475873.1 | p_Proteobacteria; c_Gammaproteobacteria; o_Pseudomonadales; f_Pseudomonadaceae; g_Pseudomonas; s_Pseudomonas koreensis | ST07 | Voltri | Rhizosphere soil |  |  |
| AJ628163.1 | p_Proteobacteria; c_Gammaproteobacteria; o_Pseudomonadales; f_Pseudomonadaceae; g_Pseudomonas; s_Pseudomonas pseudoalcaligenes | ST12, ST07 | Voltri, CVA | Guadalquivir River |  |  |
| AM778014.1 | p_Proteobacteria; c_Gammaproteobacteria; o_Xanthomonadales; f_Xanthomonadaceae; g_Silanimonas; s_uncultured bacterium | BdJ | CVA | Serpentinizing site | KX348537.1  (99%, 98%) | Denitrifying process |
| KF511881.1 | p_Proteobacteria; c_Gammaproteobacteria; o_Xanthomonadales; f_Xanthomonadaceae; g_Silanimonas; s_uncultured bacterium | BdJ | Voltri, CVA, The Cedars | Chromium contaminated soil |  |  |
| FJ405365.1 | p_Proteobacteria; c_Gammaproteobacteria; o_Xanthomonadales; f_Xanthomonadaceae; g_Stenotrophomonas; s_Xanthomonas sp. SPf | ST12 | CVA | Sweet potato plants |  |  |
| KC238323.1 | p_Proteobacteria; c_Gammaproteobacteria; o_Xanthomonadales; f_JTB255 marine benthic group; g_uncultured bacterium; s_uncultured bacterium | ST12 | Lost-City | Bio-filtration of seawater |  |  |

**^(*)^** Accession number of representative sequences of OTUs

**^(1)^** This OTU was also abundantly detected in CROMO. (Twing et al., 2017)

# References

Brazelton, W. J., Schrenk, M. O., Kelley, D. S., and Baross, J. A. (2006). Methane-and sulfur-metabolizing microbial communities dominate the Lost City Hydrothermal Field ecosystem. *Appl. Environ. Microbiol.* 72, 6257–6270. doi:10.1128/AEM.00574-06.

Daae, F. L., Økland, I., Dahle, H., Jørgensen, S. L., Thorseth, I. H., and Pedersen, R. B. (2013). Microbial life associated with low-temperature alteration of ultramafic rocks in the Leka ophiolite complex. *Geobiology* 11, 318–339. doi:10.1111/gbi.12035.

Pisapia, C., Gérard, E., Gérard, M., Lecourt, L., Lang, S. Q., Pelletier, B., et al. (2017). Mineralizing filamentous bacteria from the Prony Bay Hydrothermal Field give new insights into the functioning of serpentinization-based subseafloor ecosystems. *Front. Microbiol.* 8. doi:10.3389/fmicb.2017.00057.

Postec, A., Quéméneur, M., Bes, M., Mei, N., Benaïssa, F., Payri, C., et al. (2015). Microbial diversity in a submarine carbonate edifice from the serpentinizing hydrothermal system of the Prony Bay (New Caledonia) over a 6-year period. *Front. Microbiol.* 6, 1–19. doi:10.3389/fmicb.2015.00857.

Quéméneur, M., Bes, M., Postec, A., Mei, N., Hamelin, J., Monnin, C., et al. (2014). Spatial distribution of microbial communities in the shallow submarine alkaline hydrothermal field of the Prony Bay, New Caledonia. *Environ. Microbiol. Rep.*, 665–674. doi:10.1111/1758-2229.12184.

Quéméneur, M., Palvadeau, A., Postec, A., Monnin, C., Chavagnac, V., Ollivier, B., et al. (2015). Endolithic microbial communities in carbonate precipitates from serpentinite-hosted hyperalkaline springs of the Voltri Massif (Ligurian Alps, Northern Italy). *Environ. Sci. Pollut. Res.* 22, 13613–13624. doi:10.1007/s11356-015-4113-7.

Schrenk, M. O., Kelley, D. S., Bolton, S. A., and Baross, J. A. (2004). Low archaeal diversity linked to subseafloor geochemical processes at the Lost City Hydrothermal Field, Mid-Atlantic Ridge. *Environ. Microbiol.* 6, 1086–1095. doi:10.1111/j.1462-2920.2004.00650.x.

Suzuki, S., Ishii, S., Wu, A., Cheung, A., Tenney, A., Wanger, G., et al. (2013). Microbial diversity in The Cedars, an ultrabasic, ultrareducing, and low salinity serpentinizing ecosystem. *PNAS* 110, 15336–15341. doi:10.1073/pnas.1302426110.

Tiago, I., and Veríssimo, A. (2013). Microbial and functional diversity of a subterrestrial high pH groundwater associated to serpentinization. *Environ. Microbiol.* 15, 1687–1706. doi:10.1111/1462-2920.12034.

Twing, K. I., Brazelton, W. J., Kubo, M. D. Y., Hyer, A. J., Cardace, D., Hoehler, T., et al. (2017). Serpentinization-influenced groundwater harbors extremely low diversity microbial communities adapted to high pH. *Front. Microbiol.* 8, 308. doi:10.3389/FMICB.2017.00308.
