# Supplementary material for: Diversity of Rare and Abundant Prokaryotic Phylotypes in the Prony Hydrothermal Field and Comparison with Other Serpentinite-Hosted Ecosystems
Source: Front Microbiol. 2018 Feb 6;9:102. doi: 10.3389/fmicb.2018.00102 (PMC5808123; doi:10.3389/fmicb.2018.00102)
Supplement: Supplementary file 6 [file Table_6.DOCX]

Supplementary Table 6. Taxonomy details of archaeal OTUs shared at least by one sample from PHF (this study, Pisapia et al., 2017; Postec et al., 2015; Quéméneur et al., 2014) and by one sample from another serpentinizing system among: Cabeço de Vide Aquifer (Tiago and Veríssimo, 2013), The Cedars (Suzuki et al., 2013), Voltri ophiolite (Quéméneur et al., 2015), Leka ophiolite (Daae et al., 2013) and Lost-City (Brazelton et al., 2006; Schrenk et al., 2004). If the representative sequence of an OTU was isolated in a serpentinizing ecosystem, the last two columns indicate the closest relative of this sequence, identified in another kind of environment (not influenced by serpentinization reaction).

| **OTUs and their distribution in studied serpentinizing systems** | | | | | **Closest relative in ecosystems that are not reported as serpentinizing sites** | |
| --- | --- | --- | --- | --- | --- | --- |
| **Genbank ID^(*)^** | **Taxonomy (p_Phylum; c_Class; o_Order; f_Family; g_Genus; s_Species)** | **PHF sites** | **Other ecosystems** | **Representative sequence source** | **Genbank ID (coverage, identity)** | **Environment** |
| FJ791591.1 | p_Euryarchaeota; c_Methanomicrobia; o_Methanosarcinales; f_Methanosarcinaceae;  g_ANME-3; s_uncultured archaeon | ST12, BdJ | Lost-City | Serpentinizing site | DQ228615.1  (98%, 95%) | sulfide-microbial incubator |
| KC574884.1 | p_Euryarchaeota; c_Methanomicrobia; o_Methanosarcinales; f_Methermicoccaceae;  g_Methermicoccus; s_uncultured bacterium | ST12, ST09, ST07, BdJ | The Cedars | Serpentinizing site | AY835421.2  (99%, 89%) | hydrothermal sediment |
| JX507272.1 | p_Euryarchaeota; c_Thermococci; o_Thermococcales; f_Thermococcaceae;  g_Thermococcus; s_uncultured archaeon | ST12 | Lost-City | Guaymas Basin Hydrothermal Vent |  |  |
| AY505046.1 | p_Thaumarchaeota; c_Marine Group I; o_uncultured archaeon; f_uncultured archaeon;  g_uncultured archaeon; s_uncultured archaeon | ST09 | Lost-City | Serpentinizing site | KP204688.1  (100%, 97%) | Orca Basin South hypersaline sediment |

**^(*)^** Accession number of representative sequences of OTUs

# References

Brazelton, W. J., Schrenk, M. O., Kelley, D. S., and Baross, J. A. (2006). Methane-and Sulfur-Metabolizing Microbial Communities Dominate the Lost City Hydrothermal Field Ecosystem. *Appl. Environ. Microbiol.* 72, 6257–6270. doi:10.1128/AEM.00574-06.

Daae, F. L., Økland, I., Dahle, H., Jørgensen, S. L., Thorseth, I. H., and Pedersen, R. B. (2013). Microbial life associated with low-temperature alteration of ultramafic rocks in the Leka ophiolite complex. *Geobiology* 11, 318–339. doi:10.1111/gbi.12035.

Pisapia, C., Gérard, E., Gérard, M., Lecourt, L., Lang, S. Q., Pelletier, B., et al. (2017). Mineralizing Filamentous Bacteria from the Prony Bay Hydrothermal Field Give New Insights into the Functioning of Serpentinization-Based Subseafloor Ecosystems. *Front. Microbiol.* 8. doi:10.3389/fmicb.2017.00057.

Postec, A., Quéméneur, M., Bes, M., Mei, N., Benaïssa, F., Payri, C., et al. (2015). Microbial diversity in a submarine carbonate edifice from the serpentinizing hydrothermal system of the Prony Bay (New Caledonia) over a 6-year period. *Front. Microbiol.* 6, 1–19. doi:10.3389/fmicb.2015.00857.

Quéméneur, M., Bes, M., Postec, A., Mei, N., Hamelin, J., Monnin, C., et al. (2014). Spatial distribution of microbial communities in the shallow submarine alkaline hydrothermal field of the Prony Bay, New Caledonia. *Environ. Microbiol. Rep.*, 665–674. doi:10.1111/1758-2229.12184.

Quéméneur, M., Palvadeau, A., Postec, A., Monnin, C., Chavagnac, V., Ollivier, B., et al. (2015). Endolithic microbial communities in carbonate precipitates from serpentinite-hosted hyperalkaline springs of the Voltri Massif (Ligurian Alps, Northern Italy). *Environ. Sci. Pollut. Res.* 22, 13613–13624. doi:10.1007/s11356-015-4113-7.

Schrenk, M. O., Kelley, D. S., Bolton, S. A., and Baross, J. A. (2004). Low archaeal diversity linked to subseafloor geochemical processes at the Lost City Hydrothermal Field, Mid-Atlantic Ridge. *Environ. Microbiol.* 6, 1086–1095. doi:10.1111/j.1462-2920.2004.00650.x.

Suzuki, S., Ishii, S., Wu, A., Cheung, A., Tenney, A., Wanger, G., et al. (2013). Microbial diversity in The Cedars, an ultrabasic, ultrareducing, and low salinity serpentinizing ecosystem. *PNAS* 110, 15336–15341. doi:10.1073/pnas.1302426110.

Tiago, I., and Veríssimo, A. (2013). Microbial and functional diversity of a subterrestrial high pH groundwater associated to serpentinization. *Environ. Microbiol.* 15, 1687–1706. doi:10.1111/1462-2920.12034.
