# Supplementary figures and images for: Diversity of Rare and Abundant Prokaryotic Phylotypes in the Prony Hydrothermal Field and Comparison with Other Serpentinite-Hosted Ecosystems
Source: Front Microbiol. 2018 Feb 6;9:102. doi: 10.3389/fmicb.2018.00102 (PMC5808123; doi:10.3389/fmicb.2018.00102)

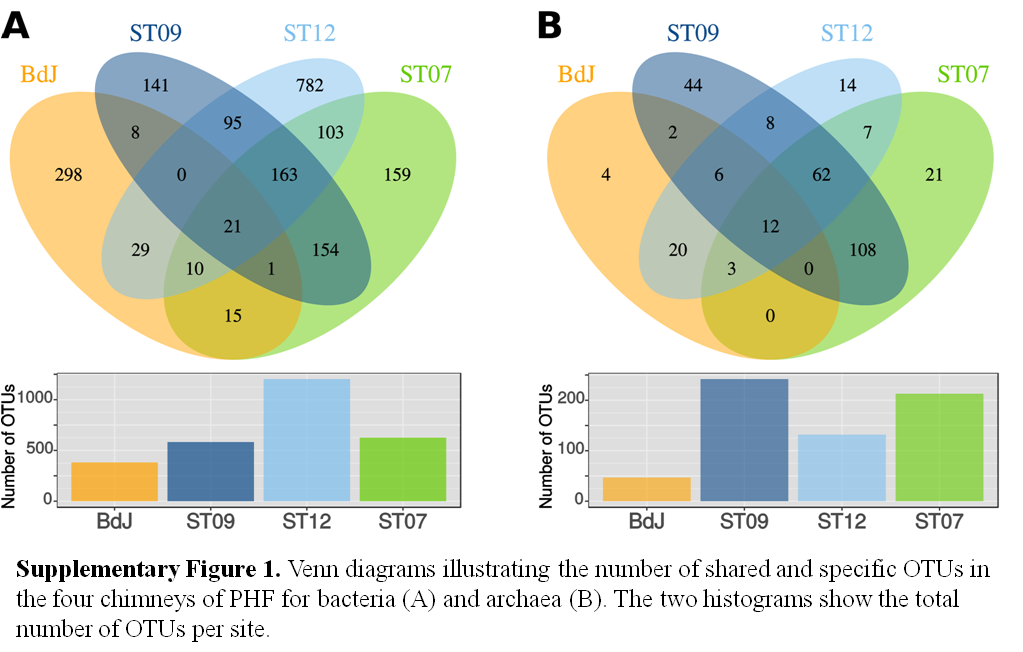

Supplement: Supplementary file 7 [file Image_1.PNG]

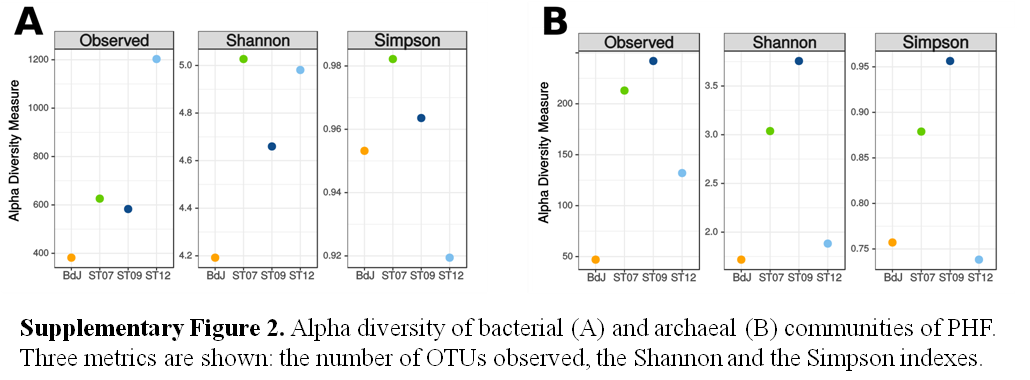

Supplement: Supplementary file 8 [file Image_2.PNG]

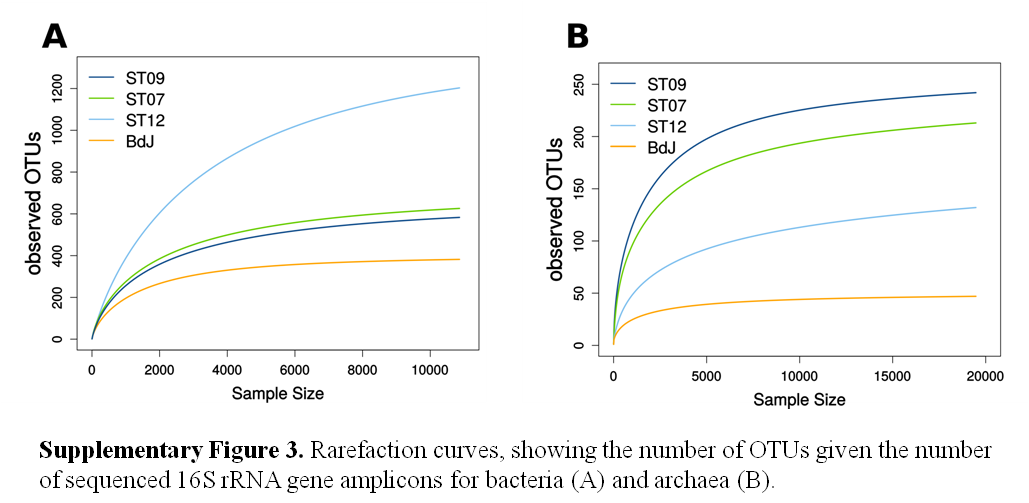

Supplement: Supplementary file 9 [file Image_3.PNG]
